# *Staphylococcus aureus* Quorum Regulator SarA Targeted Compound, 2-[(Methylamino)methyl]phenol Inhibits Biofilm and Down-Regulates Virulence Genes

**DOI:** 10.3389/fmicb.2017.01290

**Published:** 2017-07-11

**Authors:** P. Balamurugan, V. Praveen Krishna, D. Bharath, Raajaraam Lavanya, Pothiappan Vairaprakash, S. Adline Princy

**Affiliations:** ^1^Quorum Sensing Laboratory, Centre for Research on Infectious Diseases, School of Chemical and Biotechnology, SASTRA University Thanjavur, India; ^2^Department of Chemistry, School of Chemical and Biotechnology, SASTRA University Thanjavur, India

**Keywords:** *Staphylococcus aureus*, SarA, antibiotics, quorum sensing, RT-PCR, multidrug resistance, gene expression

## Abstract

*Staphylococcus aureus* is a widely acknowledged Gram-positive pathogen for forming biofilm and virulence gene expressions by quorum sensing (QS), a cell to cell communication process. The quorum regulator SarA of *S. aureus* up-regulates the expression of many virulence factors including biofilm formation to mediate pathogenesis and evasion of the host immune system in the late phases of growth. Thus, inhibiting the production or blocking SarA protein might influence the down-regulation of biofilm and virulence factors. In this context, here we have synthesized 2-[(Methylamino)methyl]phenol, which was specifically targeted toward the quorum regulator SarA through *in silico* approach in our previous study. The molecule has been evaluated *in vitro* to validate its antibiofilm activity against clinical *S. aureus* strains. In addition, antivirulence properties of the inhibitor were confirmed with the observation of a significant reduction in the expression of representative virulence genes like *fnbA, hla* and *hld* that are governed under *S. aureus* QS. Interestingly, the SarA targeted inhibitor showed negligible antimicrobial activity and markedly reduced the minimum inhibitory concentration of conventional antibiotics when used in combination making it a more attractive lead for further clinical tests.

## Introduction

*Staphylococcus aureus* is an opportunistic pathogen widely acknowledged for its various biofilm-related infections (Lister and Horswill, 2014). The prevalence of methicillin-resistant *Staphylococcus aureus* (MRSA) infections is increasing evidently and continues to be a menace in healthcare (Lowy, 2003; Chambers and Deleo, 2009; Liu, 2009). The constraint in perfusion of antimicrobial agents into biofilm pose threat to treat biofilm-related infections (Singh et al., 2016). Moreover, cell to cell communication in bacteria which is referred as quorum sensing (QS) enables them to reach a threshold in their population and express various virulence factors not limited only to biofilm formation. Thus, the attenuation of QS by modulating transcriptional regulators to prevent biofilm formation and inhibit virulence factors offers an attractive strategy to treat such infections (Hentzer and Givskov, 2003). More importantly, little or no selective pressure in QS inhibitory approach which does not confer resistance is an added benefit when compared to the conventional antimicrobials (Defoirdt et al., 2010). However, in spite of these claims the future of QS targeted medical therapy as a sole alternative to conventional antibiotics is uncertain at the present scenario (Sully et al., 2014). Hence, considering the combined use of antiquorum compounds and conventional antibiotics would be more rationale to address bacterial infections in a superior way.

Out of the identified and established QS mechanisms in *S. aureus*, Sar locus governs the inception of *agr* locus and in addition independently triggers some of the virulence genes expression (Chien and Cheung, 1998; Chien et al., 1999). Particularly, SarA is an important transcriptional regulator that activates *agr* operon and also modulates the expression of many virulence genes (Dunman et al., 2001). For instance, cytotoxic virulence factors like α and δ hemolysins are coded by *hla* and *hld* genes, respectively, and play a vital role in severe Staphylococcal infections. Also, *SarA* encompasses an open reading frame of 375 bp that codes for DNA-binding protein which is essential for the optimal transcription of RNA III (Cheung and Manna, 2005). Thus, interference with SarA activity signifies an alternative therapeutic approach to Staphylococcal infections. In this context, we proposed to screen molecules against the quorum regulator, SarA in our earlier *in silico* structure-assisted screening approach (Arya and Princy, 2013) and had identified several lead molecules. One amongst them, 2-[(Methylamino)methyl]phenol termed as SarABI-12 with a molecular weight of 137.18, ALogP of 1.125, 2 rotatable bonds, 2 hydrogen acceptors and 2 hydrogen donors exhibited LibDock binding score of 53.8786 to quorum regulator of *S. aureus*, SarA, at residues Glu-89 and Arg-90 (Arya and Princy, 2013). The present study involves the synthesis of 2-[(Methylamino)methyl]phenol and further validation of its antibiofilm activity and down-regulation of virulence gene expressions. As we mentioned earlier, combined use of antiquorum compounds and conventional antibiotics were carried out which showed good results. To our knowledge, this is the first time 2-[(Methylamino)methyl]phenol has been evaluated and reported for its antibiofilm and antivirulence activity.

## Materials and Methods

### Reagents and Chemicals

Microbiological media and antibiotic disks were bought from HiMedia, Mumbai. Antibiotic powders were purchased from SRL fine chemicals, India. Chemical reagents for compound synthesis were purchased from Sigma–Aldrich, United States. LIVE/DEAD BacLight Bacterial Viability Kit (L-7012) was purchased from Thermo Fisher Scientific, United States. Qiagen RNeasy Mini kit was purchased from Qiagen, Germany and iScript cDNA synthesis kit was purchased from Bio-Rad, Hercules, CA, United States. SYBR Green PCR Master Mix was purchased from Thermo Fisher Scientific, United States. Primers used in this study were from Integrated DNA Technologies, United States.

### Chemical Synthesis of 2-[(Methylamino)methyl]phenol

**FIGURE 1:**
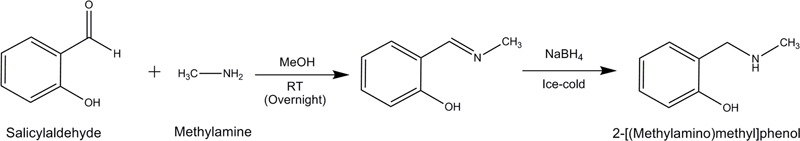
Synthesis scheme for 2-[(Methylamino)methyl]phenol.

The scheme of chemical synthesis is provided in **Figure 1**. Briefly, Salicylaldehyde (5 mmol) and methylamine (7 mmol) were stirred in methanol (5 ml) at room temperature overnight. The intermediate imine formed was reduced to amine by the addition of sodium borohydride (5 mmol) at ice-cold condition. The reaction mixture was stirred continuously at room temperature for an overnight period. Further, the reaction mixture was diluted with water and extracted thrice with dichloromethane. The organic phases were pooled together, dried over anhydrous sodium sulfate and evaporated to dryness under reduced pressure in a rotary evaporator. The crude product was purified on a silica column with a methanol: DCM system. The purity and identity of the recovered product were supported by ^1^H-NMR, ^13^C-NMR and FTIR spectroscopy.

### Bacterial Strains and Culture Conditions

Two clinical isolates of *S. aureus* (P1966 and AB459) were received from a collaborator at JSS Medical College, Mysore, India. Received strains were cultured in mannitol salt agar to ascertain the viability and identity of *S. aureus*. Pure colonies obtained were maintained as glycerol stocks in -80°C. SarA deletion mutant, i.e., *S. aureus* ALC637 strain (ΔsarA::Tn917LTV1) was a kind gift from Professor Christiane Wolz. Strains were grown in tryptic soy broth (TSB) supplemented with 0.5% glucose and incubated at 37°C under the stationary condition for subsequent assays.

### Antibiotic Susceptibility Testing for Drug Resistance

*Staphylococcus aureus* isolates were cultured to early exponential phase (1.4 × 10^7^ CFU/ml). Antibiotic susceptibility assay was performed to determine the drug resistance of *S. aureus* to various antibiotics that are commonly administered for *S. aureus* infections (Supplementary Table S1). A bacterial suspension of each strain was swabbed onto Mueller-Hinton agar plates and allowed to dry for 10 min. Antibiotic disks were placed on the plates and incubated for 24 h at 37°C. After the incubation period, the zone of inhibition diameter was measured and interpreted using the standard chart.

### Minimum Inhibitory Concentration (MIC) and Minimum Biofilm Inhibitory Concentration (MBIC) Determination

The overnight bacterial culture was inoculated in 10 ml of sterile TSB media and incubated at 37°C for 6 h to reach log phase culture. Ten microlitre of the diluted inoculum (1:200 dilutions, approximately containing 2.1 × 10^6^ CFU/ml) was inoculated into 96 well polystyrene microtitre plates containing different concentrations of the compound (0.075–1500 μM in twofold dilutions) in TSB supplemented with 0.5% (w/v) glucose. The plates were incubated at 37°C for 24 h and growth was measured at OD_595_. The lowest concentration of the compound that prevents a visible growth was considered as minimum inhibitory concentration (MIC).

Similarly, biofilm inhibition was determined with another set of 96 well microtitre plate treated as above. Briefly, the planktonic cells were removed after the incubation period, and the wells were washed with 200 μl PBS twice to remove unbound or loosely adhered cells. The cells were then stained with 100 μl of 0.2% crystal violet for 20 min. After removing the excess stain by washing, the plates were air dried. The bound crystal violet was eluted with 33% acetic acid and the optical reading was taken at OD_595_ in ELISA plate reader (BioRad i-Mark, Japan). Minimum biofilm-inhibiting concentration (MBIC) is the lowest concentration of the compound in which the formation of biofilm is limited to a level ≥90% (MBIC_90_) or ≥50% (MBIC_50_) in comparison with the untreated control cultures. All assays were carried out in triplicates.

### Visualization of Biofilm Inhibition by Confocal Microscopy Imaging

Biofilm inhibition was visually confirmed by confocal microscopy imaging. Clean sterile glass slides were placed in 15 ml of TSB medium in the absence (positive control) and the presence of the test compound (0.6, 1.25, and 2.5 μM) in a 50 ml centrifuge tube. One percent (100 μl) of the diluted bacterial culture (2.1 × 10^6^ CFU/ml) was added and incubated for 6 h at 37°C. After 6 h, the slides were rinsed with sterile PBS to remove non-adherent planktonic cells and stained with *Bac*Light Bacterial Viability Kit (L7012) as per the kit protocol. Two and three-dimensional images were captured using a confocal laser scanning microscope with a 40× objective lens to show biofilm distribution (Olympus FLUOVIEW, FV1000).

### Quantification of Gene Expression Using qRT-PCR

*Staphylococcus aureus* isolates (P1966 and AB459) were grown in TSB in the presence (1.25 μM) and absence of test compound for 6 and 24 h at 37°C. After the incubation period, the walls of the test tubes were scrapped with a sterile cell scraper to bring the adhered cells into suspension. The total cell population (planktonic and biofilm) were harvested by centrifugation from grown cultures (0.5 ml) at log phase (6 h) and stationary phase (24 h) and immediately stored at -80°C. RNA was isolated using a Qiagen RNeasy Mini kit in accordance with the manufacturer’s instructions. RNA concentrations were determined by optical density measurements in a spectrophotometer at OD_260_ (Thermo Scientific NanoDrop, United States). cDNA synthesis was carried out using the iScript cDNA synthesis kit according to the manufacturer’s instructions. Briefly, the reaction mixture was incubated for annealing at 25°C for 5 min, extension at 42°C for 30 min and inactivation of samples at 85°C for 5 min.

qRT-PCR was used to assess the transcriptional expression levels of *SarA* in the bacterial strains taken and down-regulation of representative virulence genes (*RNAIII, fnbA, hla* and *hld*) when treated with the compound. Sequences of primers used in this study are furnished in **Table 1**. The reaction mixture in a total volume of 20 μl, consisted 10 μl 2× SYBR Green PCR Master Mix, forward and reverse primers (1 μl each), 4 μl of nuclease free water and 4 μl of 20× diluted cDNA. PCR conditions included an initial denaturation at 95°C for 2 min, followed by 40 cycles of denaturation (95°C for 15 s), annealing (55.8°C for 15 s), and extension (72°C for 20 s). To ensure the samples were free from contamination, negative controls containing nuclease-free water instead of cDNA were run in parallel. The relative gene expression was analyzed using the 2^-ΔΔCT^ method with 16S rRNA as a reference gene.

**Table 1:** Primers used in this study.

Gene	Forward primer (5′ to 3′)	Reverse primer (5′ to 3′)	Reference
*16S rRNA*	TGATCCTGGCTCAGGATGA	TTCGCTCGACTTGCATGTA	Sully et al., 2014
*SarA*	TCTTGTTAATGCACAACAACGTAA	TGTTTGCTTCAGTGATTCGTTT	Abdelhady et al., 2014
*RNAIII*	AATTAGCAAGTGAGTAACATTTGCTAGT	GATGTTGTTTACGATAGCTTACATGC	Sully et al., 2014
*fnbA*	ACAAGTTGAAGTGGCACAGCC	CCGCTACATCTGCTGATCTTGTC	Vaudaux et al., 2002
*hla*	ACAATTTTAGAGAGCCCAACTGAT	TCCCCAATTTTGATTCACCAT	Sully et al., 2014
*hld*	AAGAATTTTTATCTTAATTAAGGAAGGAGTG	TTAGTGAATTTGTTCACTGTGTCGA	Alfatemi et al., 2014

### Cytotoxicity Assay

The MTT assay was carried out to determine any cytotoxicity effect of 2-[(Methylamino)methyl]phenol. Eagle’s Minimum Essential Medium supplemented with non-essential amino acids, 10% fetal bovine serum and penicillin-streptomycin (100 U/ml–100 μg/ml)) was used as culture medium. In brief, 200 μl of viable Human epithelial type 2 (HEp-2) cells in a concentration of 1.5 × 10^4^ cells/ml were seeded in 96 well tissue culture plates. Varying concentrations (1.25, 12.5, and 125 μM) of the synthesized compound were added to each well when cells reached 80% confluence. The plate was incubated at 37°C for 24 h with 5% CO_2._ Further 20 μl of MTT solution (5 mg/ml) were added to each well and incubated for 3 h. Following the incubation period, 200 μl of DMSO was added to the wells and OD was taken at OD_570_. The percentage cell viability was calculated with reference to the untreated control cells using the below formula.

Percentage cell viability

$$\frac{\text{OD}\;\text{of}\;\text{Drug-treated}\;\text{sample}-\text{OD}\;\text{of}\;\text{Blank}}{\text{OD}\;\text{of}\;\text{Control}-\text{OD}\;\text{of}\;\text{Blank}}\times 100$$

### Combinatorial Studies

Combinatorial studies were carried out between selected antibiotics that are commonly used for Staphylococcal infections and the test compound 2-[(Methylamino)methyl]phenol at the MBIC_50_ concentration. The *S. aureus* strains (P1966 and AB459) were cultured in TSB along with test compound at the fixed MBIC concentration, 1.25 μM and varying concentration of antibiotics in twofold dilutions at 37°C for 24 h at the stationary condition. At the end of incubation, absorbance was measured at OD_595_ and MIC was noted.

### Statistical Analysis

Statistical analysis was carried out using GraphPad Prism software version 6.05 (GraphPad Software Inc., San Diego, CA, United States). One-way ANOVA and multiple comparisons were carried out to test the significance. The minimum level of significance was set at *p* ≤ 0.05. All the assays were carried out in triplicates and the results were expressed as mean ± SD.

## Results

### Synthesis of 2-[(Methylamino)methyl]phenol

2-[(Methylamino)methyl]phenol was synthesized as shown in the reaction scheme (**Figure 1**). The obtained compound had a yield of 82%, golden yellow in appearance. ^1^H-NMR (300 MHz, CDCl_3_) δ2.48 (s, 3H), 3.97(s, 2H), 6.75–6.80 (m, 1H), 6.83 (d, *J* = 8.1 Hz, 1H), 6.99 (d, *J* = 7.2 Hz, 1H), 7.14–7.29 (m, 1H). ^13^C-NMR (75 MHz, CDCl_3_) 35.1, 54.5, 122.2, 128.5, 116.3, 118.9, 128.8, 158.3. IR (Neat) 3430.7, 3001.7, 1646.0, 1434.8, 1411.6, 1261.2, and 1023.1 cm^-1^. Spectra of ^1^H-NMR, ^13^C-NMR and FTIR are provided in **Supplementary Figures S1–S3**, respectively.

### Antibiotic Susceptibility Testing for Drug Resistance

The resistance pattern to various conventionally used antibiotics against *S. aureus* infections was shown in Supplementary Table S1. The clinical isolates exhibited multidrug resistance to most of the tested antibiotics that include gentamicin, azithromycin, erythromycin, co-trimoxazole, ciprofloxacin, doxycycline, methicillin, oxacillin, cloxacillin, cefuroxime, cephalexin, vancomycin and trimethoprim.

### Antibacterial and Antibiofilm Activity

**FIGURE 2:**
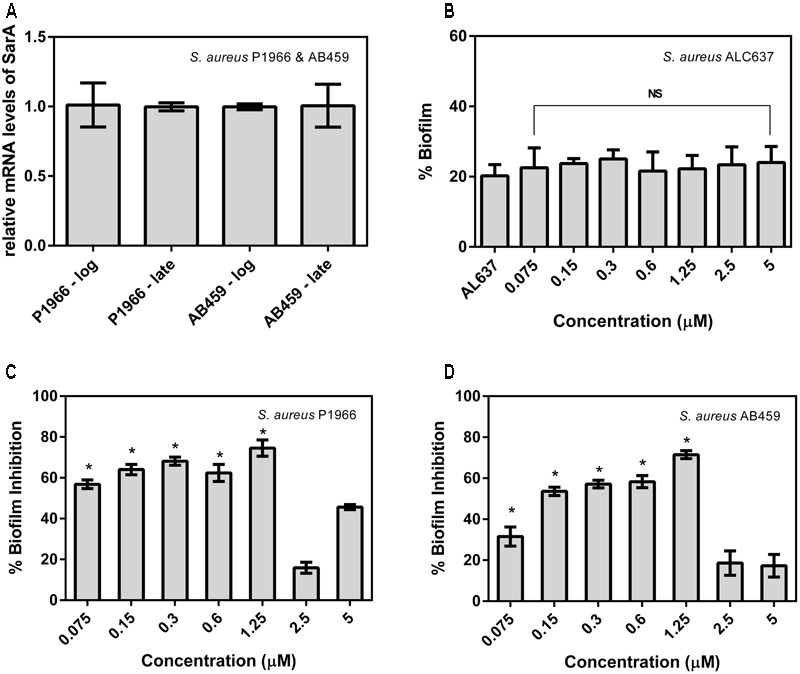
Percentage biofilm formed and inhibited in *Staphylococcus aureus.* **(A)** Expression levels of *SarA* in the bacterial strains taken in this study at the log and late phases of growth. **(B)** Percentage biofilm formation of *SarA* mutant ALC637 (ΔsarA::795 Tn917LTV1) on treatment and no treatment with 2-[(Methylamino)methyl]phenol by crystal violet method. **(C,D)** Percentage biofilm inhibition in clinical *S. aureus* isolates P1966 and AB459 by 2-[(Methylamino)methyl]phenol. All the assays were done in triplicates and the values were expressed as mean ± SD. ^∗^Indicates significantly different (*p* ≤ 0.05) when compared to untreated (control) with 2-[(Methylamino)methyl]phenol. NS denotes not significant (*P* > 0.05).

The expression level of SarA in the clinical isolates taken in this study was evaluated by qRT-PCR which confirms that the strains express SarA in the log and late phases of growth and is valid for further gene expression studies with the drug at corresponding phases (**Figure 2A**). The SarA deletion mutant ALC637 did not show any significant difference in biofilm when treated with different concentrations of the SarA targeted compound, 2-[(Methylamino)methyl]phenol (**Figure 2B**). Antibiofilm activity was evaluated for the SarA target-specific compound 2-[(Methylamino)methyl]phenol against the SarA positive clinical *S. aureus* strains and the results were presented in **Figures 2C,D**. Significant biofilm inhibition (>70%) was observed at a concentration of 1.25 μM. Moreover, 2-[(Methylamino)methyl]phenol did not show any antibacterial activity in the entire test range of concentrations up to 1500 μM. The compound did not exhibit disruption of preformed biofilm of both the strains (data not shown).

### Visualization of Biofilm Inhibition by Confocal Microscopy Imaging

**FIGURE 3:**
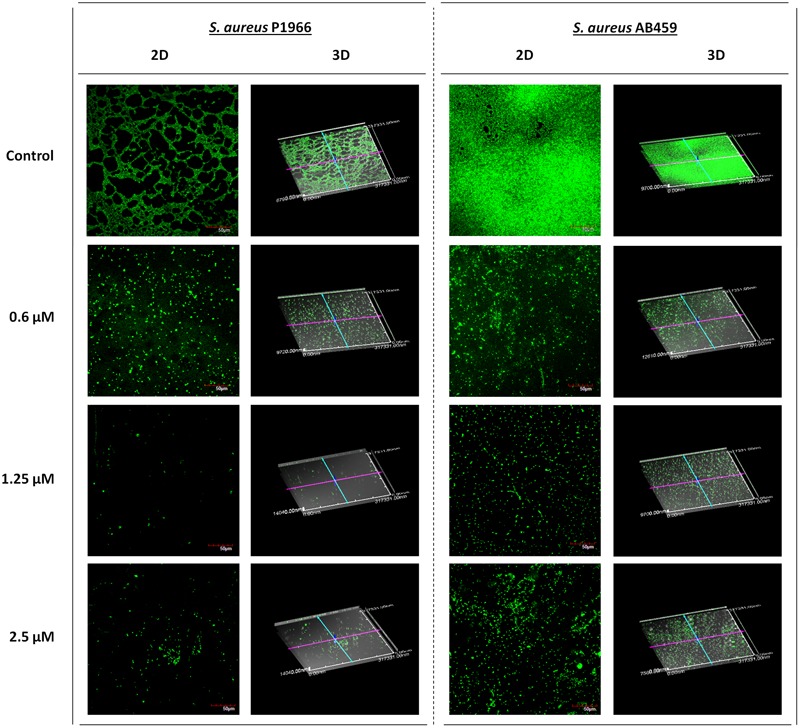
Confocal laser scanning microscopy imaging. Representative images showing the biofilm inhibition effects of 2-[(Methylamino)methyl]phenol at varying concentrations on *Staphylococcus aureus* P1966 and AB459 during log phase of growth (6 h). The results were in concordance with microtitre plate quantitative assays where biofilm inhibition was more at 1.25 μM. Scale bar in images represents 50 μm.

Visual confirmation of the biofilm inhibition by the compound was checked by Live/Dead staining in a confocal laser scanning microscope (**Figure 3**). The maximum biofilm inhibited concentration of the compound in microtitre plate assays (1.25 μM), also showed more inhibition in the captured images compared to that of the lower (0.6 μM) and higher (2.5 μM) concentrations. The results were in concordance with our crystal violet biofilm inhibition studies. The absence of dead cells (presented by red fluorescence) in Live/Dead stained images indicate the exclusion of any antibacterial activity possessed by the compound.

### Gene Expression Profiling Using qRT-PCR

**FIGURE 4:**
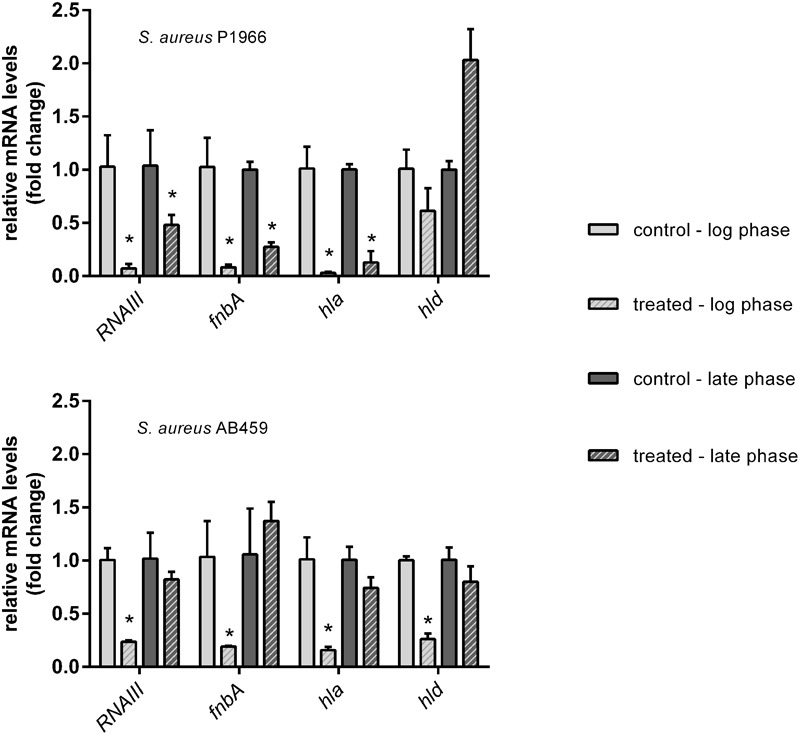
qRT-PCR analyses of representative virulence genes under the control of SarA. Downregulation of virulence genes (*RNAIII, fnbA, hla* and *hld*) in *S. aureus* P1966 and AB459 treated with 1.25 μM of 2-[(Methylamino)methyl]phenol at the log and late phases of growth are expressed a fold change by the 2^-ΔΔCT^ method. 16S rRNA was used as reference gene for data normalization. ^∗^Indicates significantly different (*p* ≤ 0.05) when compared to untreated (control) with 2-[(Methylamino)methyl]phenol.

Representative genes took in the present study (*RNAIII, fnbA, hla* and *hld*) under the control of quorum regulator SarA were down-regulated significantly except *hld* at both the log and late phases of growth in the case of *S. aureus* P1966. Whereas, in the case of *S. aureus* AB459, downregulation was more in the log growth phase compared to late growth phase (**Figure 4**).

### Cytotoxicity Assay

**FIGURE 5:**
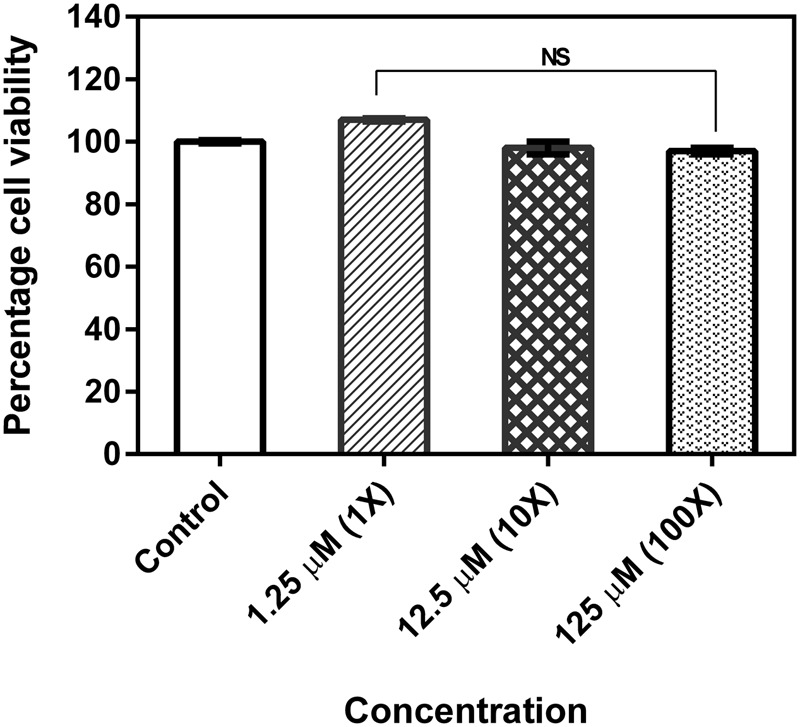
Cell viability of human epithelial type 2 (HEp-2) cells treated with 2-[(Methylamino)methyl]phenol. No significant difference between untreated and treated with varying concentrations of the test compound by MTT assay. The assay was done in triplicates and the values were expressed as mean ± SD. NS denotes not significant (*P* > 0.05).

MTT assay data showed that the drug neither inhibits the proliferation of cells nor gives toxic effect to the HEp-2 cells even after incubating the cells with the compound at its MBIC concentration. Percentage of cell viability was equal to that of control (untreated cells) even after cells were exposed to 10× and 100× MBIC concentrations of the compound (**Figure 5**).

### Combinatorial Studies

Effect of the test compound in reducing the MIC values of conventionally used antibiotics against *S. aureus* was tested by combinatorial usage of both. In the case of *S. aureus* P1966, except for Azithromycin and Cephalexin, all other antibiotics taken showed a marked reduction in MIC values when used in combination with 1.25 μM of the test compound. Whereas in the case of *S. aureus* AB459 only a few antibiotics (Cloxacillin, Cephalexin and Vancomycin) showed marked MIC reduction and other antibiotics MICs were as same as that of when used alone (**Table 2**).

**Table 2:** Minimum inhibitory concentration of antibiotics alone and in combination with the test compound.

Antibiotic	*S. aureus* P1966		*S. aureus* AB459	
	MIC of antibiotic alone (μg/ml)	MIC of antibiotic + MBIC of test compound (μg/ml)	MIC of antibiotic alone (μg/ml)	MIC of antibiotic + MBIC of test compound (μg/ml)
Gentamicin	8	0.5	32	32
Streptomycin	16	8	16	16
Azithromycin	16	16	8	8
Ciprofloxacin	500	8	8	4
Tetracycline	32	2	32	32
Doxycycline	8	1	8	4
Cloxacillin	125	1	125	4
Cephalexin	32	32	32	8
Vancomycin	500	2	250	8
Chloramphenicol	32	4	4	4

## Discussion

Ineffectual usage of antibiotics has raised multidrug resistance to most of the commonly administered drugs against bacterial infections. In *Staphylococcus aureus*, methicillin resistance and vancomycin resistance are a major advanced and persistent concern (Loomba et al., 2010). In the present study resistance to most of the current generation antibiotics were observed, especially to the aforesaid antibiotics, underscoring the importance of *S. aureus* as a potential clinical threat. Alternatives to antibiotic therapies are the need of the hour to overcome pathologies stemming from multidrug-resistant microbes.

One of the alternative approaches to overcome multidrug resistance is tantamount to pursue drugs that do not impose survival stress on the pathogenic organism. Exploration of antibiofilm and antivirulence compounds for alternative therapy is of recent focus. The importance of the quorum regulator SarA as an alternative drug target has been detailed in the introduction and to date, not much of small molecule inhibitors are reported for SarA inhibition. However, our own research group has reported previously a range of SarA inhibitors through *in silico* approach and evaluated a top-scored SarA inhibitor, 4-[(2,4-diflurobenzyl)amino] cyclohexanol (Arya and Princy, 2013; Arya et al., 2015). In this context, one of the SarA targeted inhibitor 2-[(Methylamino)methyl]phenol that was identified in our previous *in silico* study (Arya and Princy, 2013) was evaluated for biofilm inhibition and downregulation of virulence genes.

Biofilm formation is a significant event in a bacterial life cycle that offers many benefits like environmental protection from pH fluctuations, desiccation, and physical insults, while also acting as an antibiotic diffusion barrier (Paraje, 2011). Eventually, this protection mechanism of bacteria from antimicrobials becomes problematic in clinical settings as the pathogen will endure treatments and emerge as a multidrug resistant strain. Hence, inhibiting bacterial biofilm is a vital concern and here we have observed significant biofilm inhibition in a dose-dependent manner up to 1.25 μM and after that a decrease in inhibition (**Figures 2C,D**). Similar results were observed qualitatively in microscopy imaging results also (**Figure 3**). A possible explanation for this could be because of the formation of insoluble macroaggregates of the compound at higher concentrations, which had been visibly observed on the treatment wells. These insoluble macro aggregates cannot pass through the cell membrane of bacteria to interact with the SarA protein and the very few left molecules would have entered the cell thus producing a reduced/no significant effect further in higher concentrations. In addition, the absence of antibacterial effect till the tested concentration of 1500 μM suggest that the compound could be a perfect choice for combating multidrug resistance in *S. aureus*, as it will not pose any survival stress or selection pressure on the bacteria forcing it to develop resistance. No difference in biofilm when the *SarA* deletion mutant *S. aureus* ALC637 treated with 2-[(Methylamino)methyl]phenol reveals the possible action of the compound on the specific target SarA as we could observe significant biofilm inhibition in SarA expressing clinical strains.

Our gene expression studies on representative virulence genes state that 2-[(Methylamino)methyl]phenol has antivirulence effect which was evidenced from the reduced transcriptional expressions. Fibronectin binding protein (FnBP) is one of the essential virulence factors that favor *S. aureus* for its anchorage with integrin α5β1 in a variety of non-professional phagocytic cells for successful invasion (Hudson et al., 1995; Yao et al., 1995; Almeida et al., 1996). The roles of extracellular adherence protein (Eap), plasmin-sensitive protein (Pls) and wall teichoic acid (WTA) in *S. aureus* cell invasion cannot be ruled out, but the importance of these proteins pale in comparison to FnBP-mediated adhesion (Weidenmaier et al., 2004; Hussain et al., 2008, 2009). It has been established that FnBP makes a molecular bridge between the bacterial microbial surface components recognizing adhesive matrix molecules (MSCRAMMs) and the host cell β1 integrins, leading to bacterial uptake (Fowler et al., 2000). Twofold higher levels of adhesion and 20-fold higher levels of invasion have been observed in microbial cells at exponential phase in comparison with those cells at stationary phase, during a study of bovine mammary gland invasion by *S. aureus* (Lammers et al., 1999). Our results are in concordance with this study where *fnbA* transcription down-regulation is highly significant at log phase of growth as compared to the stationary phase of growth, for combating bacterial adhesion. In our present study, both the strains of *S. aureus* showed better down-regulation of α-hemolysin compared to δ-hemolysin. Staphylococcal α-hemolysin toxins are secreted as an outcome of virulence mechanisms that are under the QS control in *S. aureus* and α–toxin is believed to be involved in the phagolysosomal escape as it is a pore-forming toxin (Jarry et al., 2008). Many earlier studies have reported the role of SarA and its effect when it is mutated making it non-functional. A mutational study on SarA has shown reduced accumulation of extracellular toxins like α–toxin and phenol soluble modulins (Mrak et al., 2012). Similarly, another study showed reduced ability to induce septic arthritis and osteomyelitis in murine models of musculoskeletal infection when SarA is mutated (Abdelnour et al., 1993; Nilsson et al., 1996; Blevins et al., 2003). All these signifies the importance of Sar A role in virulence factor expressions and scores as a potential therapeutic target.

Physical barrier to antibiotics in biofilm related infections can be tackled by the use of antibiofilm agents in combination where these would resuscitate the potency of the antibiotics. The antibiofilm agent will inhibit biofilm formation and thus exposing the planktonic cells to sub-inhibitory concentration of antibiotic that can eliminate the bacterial population by easy penetration. Combined use of the test compound and conventionally used antibiotics against *S. aureus* was tested and the results showed a significant reduction in MIC values when antibiotics were used alone. This could be attributed to the fact that, reduction in biofilm by 2-[(Methylamino)methyl]phenol would have enhanced the antibiotic action by complete diffusion into the biofilms. Such a significant reduction in MIC levels possibly have a way of reducing the over usage of antibiotic concentrations in therapeutic treatments.

## Conclusion

Our experimental results prove that 2-[(Methylamino)methyl]phenol has antibiofilm and antivirulence properties. Moreover, the SarA targeted inhibitor is specific in targeting QS of *S. aureus* and does not have cytotoxicity, making it an alternative therapeutic choice over conventional antibiotics. Further, *in vivo* studies are needed to evaluate the clinical effectiveness as a drug molecule. Thus we report, to the best of our knowledge, the first observation of antibiofilm and antivirulence properties of 2-[(Methylamino)methyl]phenol against *S. aureus*.

## Author Contributions

All authors listed have made a substantial, direct and intellectual contribution to the work, and approved it for publication.

## Conflict of Interest Statement

The authors declare that the research was conducted in the absence of any commercial or financial relationships that could be construed as a potential conflict of interest.

## Supplementary Material

FIGURE S1 ^1^H-NMR spectrum of 2-[(Methylamino)methyl]phenol.

FIGURE S2 ^13^C-NMR spectrum of 2-[(Methylamino)methyl]phenol.

FIGURE S3 FTIR spectrum of 2-[(Methylamino)methyl]phenol.
