# Supplementary figures and images for: Staphylococcus aureus Quorum Regulator SarA Targeted Compound, 2-[(Methylamino)methyl]phenol Inhibits Biofilm and Down-Regulates Virulence Genes
Source: Front Microbiol. 2017 Jul 11;8:1290. doi: 10.3389/fmicb.2017.01290 (PMC5504099; doi:10.3389/fmicb.2017.01290)

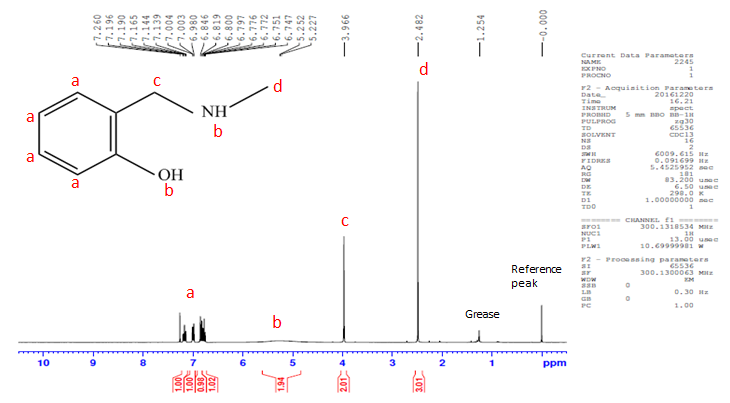

Supplement: FIGURE S1 — 1H-NMR spectrum of 2-[(Methylamino)methyl]phenol. [file Image_1.TIF]

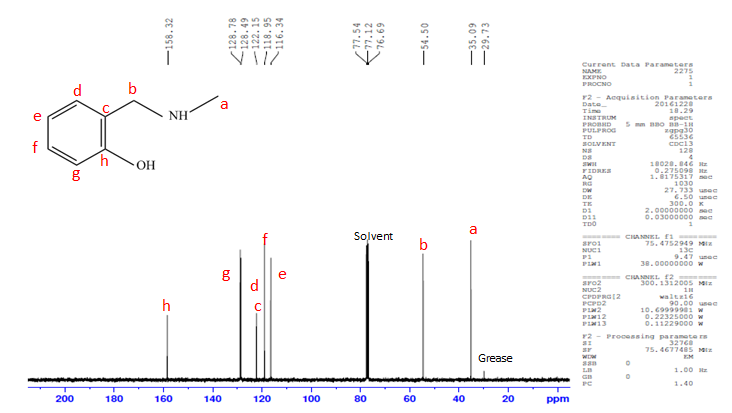

Supplement: FIGURE S2 — 13C-NMR spectrum of 2-[(Methylamino)methyl]phenol. [file Image_2.TIF]

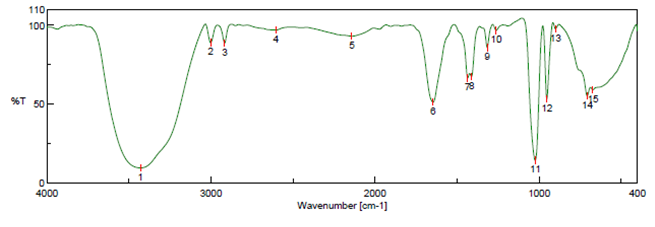

Supplement: FIGURE S3 — FTIR spectrum of 2-[(Methylamino)methyl]phenol. [file Image_3.TIF]
