# Supplementary material for: Staphylococcus aureus Quorum Regulator SarA Targeted Compound, 2-[(Methylamino)methyl]phenol Inhibits Biofilm and Down-Regulates Virulence Genes
Source: Front Microbiol. 2017 Jul 11;8:1290. doi: 10.3389/fmicb.2017.01290 (PMC5504099; doi:10.3389/fmicb.2017.01290)
Supplement: Supplementary file 4 [file Table_1.DOC]

**Table S1 Antibiotic susceptibility of selected antibiotics against *Staphylococcus aureus* strains.**

| **Antibiotic disc** | **Class** | ***Staphylococcus aureus*** | |
| --- | --- | --- | --- |
|  |  | **P1966** | **AB459** |
| Gentamicin (30 µg) | Aminoglycosides | - | - |
| Streptomycin (10 µg) | Aminoglycosides | + | + |
| Azithromycin (30 µg) | Macrolides | - | - |
| Erythromycin (10 µg) | Macrolides | - | - |
| Co-trimoxazole (25 µg) | Sulfonamides | - | - |
| Ciprofloxacin (10 µg) | Quinolones | - | - |
| Linezolid (30 µg) | Oxazolidinones | + | + |
| Tetracycline (30 µg) | Tetracyclines | + | - |
| Doxycycline (30 µg) | Tetracyclines | - | - |
| Methicillin (10 µg) | Penicillins | - | - |
| Oxacillin (5 µg) | Penicillins | - | - |
| Cloxacillin (30 µg) | Penicillins | - | - |
| Cefuroxime (30 µg) | Cephalosporins | - | - |
| Cephalexin (30 µg) | Cephalosporins | - | - |
| Vancomycin (30 µg) | Glycopeptides | - | - |
| Chloramphenicol (25 µg) | Others | + | + |
| Clindamycin (2 µg) | Others | + | + |
| Trimethoprim (30 µg) | Others | - | - |

Susceptible (+); Resistant (-)
